# A Quiet Rupture: Hemodynamically Stable Ruptured Descending Thoracic Aortic Aneurysm Presenting as Lower Respiratory Tract Infection

**DOI:** 10.7759/cureus.85375

**Published:** 2025-06-04

**Authors:** Rohan Krishna N.K., Anupama R.S., Lekhana Dayanand

**Affiliations:** 1 Emergency Medicine, Fortis International Hospital, Rajajinagar, Bengaluru, IND; 2 Emergency Medicine, Fortis International Hospital Rajajinagar, Bengaluru, IND; 3 Internal Medicine, Fortis International Hospital, Rajajinagar, Bengaluru, IND

**Keywords:** aortic emergency, contained aortic rupture, ct angiography, descending thoracic aortic aneurysm, diagnostic delay, hemothorax, high-resolution ct, lower respiratory tract infection mimic, misdiagnosis, tevar

## Abstract

The rupture of a descending thoracic aortic aneurysm (DTAA) is a rare but critical vascular emergency that requires immediate recognition and action. It usually presents as a sharp, severe pain in the chest or back; however, some individuals exhibit non-typical symptoms resembling respiratory infections, leading to misdiagnosis and delays in definitive treatment. A 63-year-old male with a history of hypertension and smoking presented with left-sided chest pain to another hospital, where he was diagnosed with unstable angina based on clinical suspicion and managed conservatively with anti-anginal medication. Over the next three days, the patient developed a persistent cough, low-grade fever, and pleuritic pain, prompting referral to our hospital, where a lower respiratory tract infection (LRTI) was considered. On arrival, he was hemodynamically stable with a systolic BP of 100 mmHg and was managed with intravenous fluids, antibiotics, and nebulizers. Chest X-ray revealed moderate left pleural effusion with tracheal deviation, and thoracic ultrasound confirmed internal echoes suggestive of hemorrhagic content. Diagnostic thoracentesis yielded hemorrhagic fluid, prompting high-resolution computed tomography (HRCT), which showed a partially thrombosed 54 mm × 49 mm saccular aneurysm of the descending thoracic aorta with left lung collapse. Despite the rupture, the patient remained hemodynamically stable, suggestive of a contained event. A subsequent computed tomography angiogram (CTA) confirmed rupture into the pleural space and was the imaging modality that established the final diagnosis. The patient underwent thoracic endovascular aortic repair (TEVAR) using a 30 mm × 30 mm × 120 mm Ankura graft, selected for its conformability and effective sealing profile in emergencies. Postoperative recovery was uneventful. A CT aortogram on day three confirmed complete exclusion of the aneurysm with no endoleak, and a follow-up chest X-ray at two weeks showed full resolution of the hemothorax. This case illustrates the diagnostic challenge posed by atypical ruptured DTAA presentations and reinforces the importance of early CTA in unexplained pleural effusions, even in stable patients. Structured post-TEVAR surveillance remains critical to ensure long-term outcomes.

## Introduction

A thoracic aortic aneurysm (TAA) is defined as a permanent and localised dilatation of the aorta, predominantly 1.5 times its normal diameter, affecting all three layers of the aortic wall. The prevalence of TAA is estimated at 5 to 10 instances per 100,000 person years, with descending TAAs (DTAAs) constituting 40% of cases [1].The risk factors for DTAA include modifiable factors such as hypertension, smoking, atherosclerosis, and non-modifiable risk factors such as syndromic and familial thoracic aortic disease [2].

Ruptured DTAA (rDTAA) is a critical vascular emergency, with mortality rates reaching up to 97% without early diagnosis and treatment [3].The classical symptoms include sudden, severe chest or back pain; however, atypical symptoms may mask early detection, leading to delayed treatment and increased mortality rate. Although hemorrhagic pleural effusion is a recognized manifestation of DTAA rupture, it is less common and usually associated with hemodynamic instability. In contrast, presentations that mimic lower respiratory tract infections (LRTIs) in hemodynamically stable patients are extremely rare and have only been reported in isolated cases [4]. Such presentations are often due to contained rupture, where hemorrhage is temporarily sealed by adjacent pleura or thrombus, thereby preserving blood pressure transiently but increasing diagnostic complexity and risk.

This rarity was evident in our patient, who remained hemodynamically stable despite an rDTAA and presented with fever, productive cough, malaise, and pleuritic chest pain - an unusual clinical picture mimicking an LRTI. Based on these findings, a preliminary diagnosis of LRTI was made, and the patient was managed with nebulised bronchodilators, intravenous fluids, antibiotics, and later intravenous proton pump inhibitors. However, despite initial stabilization, his symptoms deteriorated. A chest X-ray revealed moderate pleural effusion with tracheal deviation, prompting pleural tapping, which yielded hemorrhagic fluid, raising suspicion toward a vascular cause.

Advanced imaging with high-resolution computed tomography (HRCT) demonstrated a thrombosed saccular aneurysm with associated hemorrhage, while computed tomography angiography (CTA) confirmed the diagnosis of a contained rupture. This case reinforces the need for prompt reconsideration of diagnosis and escalation to vascular imaging when standard treatments for presumed infections fail to yield improvement [5].Delays in diagnosis can result in missed opportunities, thereby causing life-threatening complications. Thoracic endovascular aortic repair (TEVAR) has emerged as a minimally invasive and preferred alternative to open surgery for managing complicated DTAAs, offering reduced perioperative morbidity and mortality. 

## Case presentation

A 63-year-old man with a medical history of hypertension and chronic smoking presented to a local hospital with a sudden onset of left-sided chest pain radiating to the back. In the absence of ECG changes or elevated troponin levels and with known cardiovascular risk factors, a diagnosis of unstable angina was made. He was treated conservatively with anti-anginal therapy, including nitrates. A transthoracic echocardiogram performed at the referring hospital revealed concentric left ventricular hypertrophy, normal biventricular function with an ejection fraction of 57%, and no regional wall motion abnormalities. He was on regular antihypertensive therapy, including telmisartan 40 mg, an angiotensin receptor blocker (ARB). There were no details regarding the family history of hypertension, thoracic aortic disease, or aneurysmal syndromes.

Over the next three days, the patient developed low-grade fever, productive cough, and generalized malaise. With limited clinical improvement and persistent respiratory symptoms, he was referred to our center for further evaluation under the impression of an LRTI.

On arrival to our emergency department, the patient appeared conscious and oriented. He remained hemodynamically stable with a heart rate of 108 beats per minute, blood pressure of 100/80 mmHg, respiratory rate of 21 breaths per minute, oxygen saturation of 99% on room air, and a temperature of 99.5 °F. On auscultation, air entry was reduced over the left lower lung fields, with no added sounds. The initial vital parameters are detailed in Table 1.

**Table 1:** Vital signs on admission.

Parameter	Values	Range
Heart rate	100 bpm	60-100 bpm
Blood pressure	100/80 mmHg	90/60 to 140/90 mmHg
Respiratory rate	21 cpm	12-20 cpm
Temperature	99.5 °F	97.0-98.6 °F (36.1-37 °C)
Oxygen saturation (SpO2)	99% on room air	≥95% on room air

**Figure 1:**
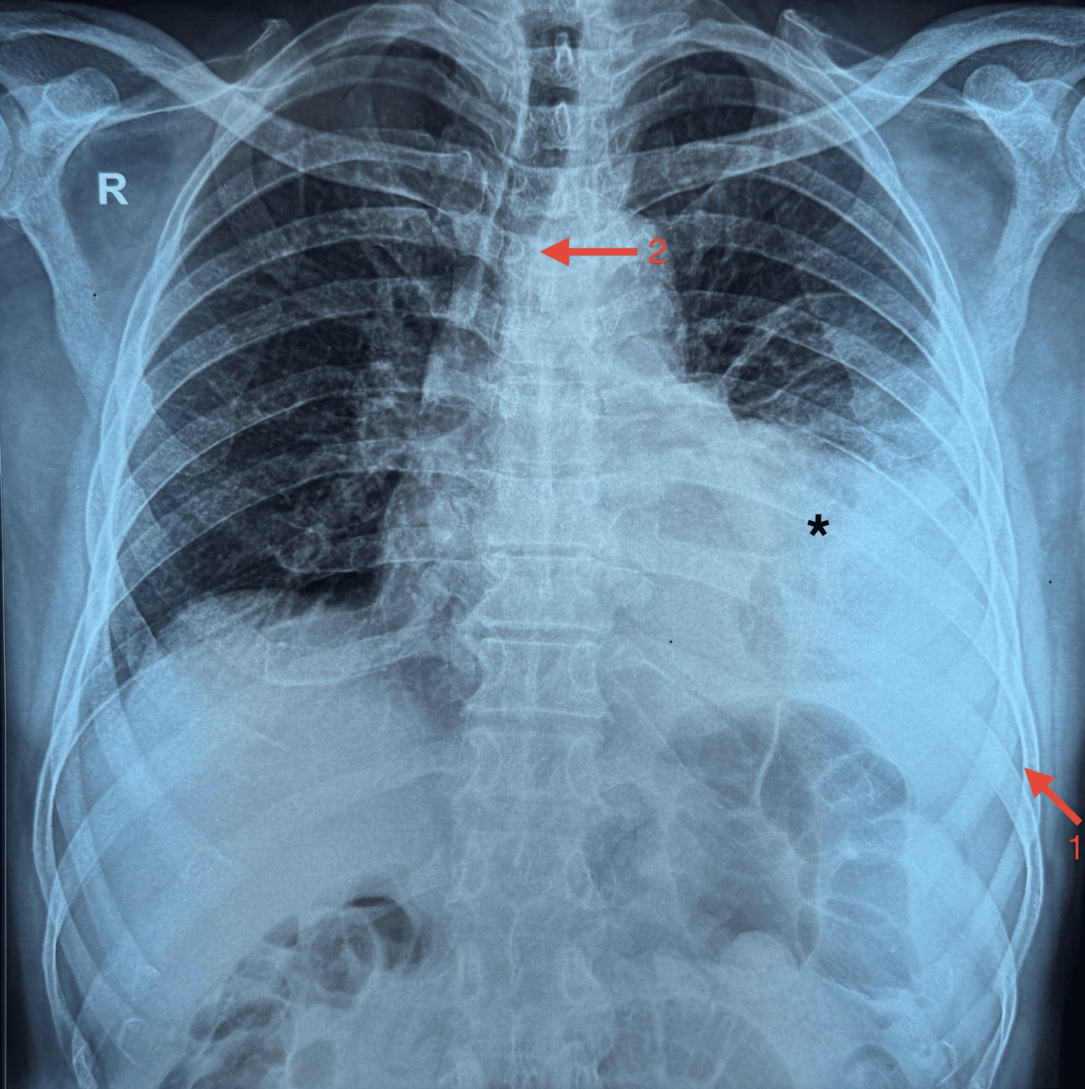
Initial chest X-ray showing blunting of the left costophrenic angle and rightward tracheal deviation, indicated by Arrow 1 and Arrow 2, respectively, suggesting significant mass effect from a left-sided pleural effusion (*).

A chest X-ray (Figure 1) was requested, which demonstrated left lower zone homogenous opacity with costophrenic angle blunting, suggesting moderate pleural effusion. A significant tracheal deviation to the right was also observed.

An ECG was done, which showed sinus tachycardia with no ischemic changes.

Baseline laboratory investigations were carried out. Hematological studies revealed mild leukocytosis with a white blood cell count of 12,000/μL, as detailed in Table 2. 

**Table 2:** Hematological parameters on admission.

Parameter	Values	Range
White blood cell (WBC) count	12.0	4.0-10.0 thou/µL
Red blood cell (RBC) count	4.51	4.5-5.5 mil/µL
Hemoglobin (Hb)	14.6	13.0-17.0 g/dL
Hematocrit (PCV)	42.7	40-50%
Mean corpuscular volume (MCV)	95.0	83.0-101.0 fL
Mean corpuscular hemoglobin (MCH)	32.3	27.0-32.0 pg
Mean corpuscular hemoglobin concentration (MCHC)	34.1	31.5-34.5 g/dL
Mentzer Index	21.1	
Red cell distribution width (RDW)	16.7	11.6-14.0%
Neutrophils	75	40-80%
Lymphocytes	20	20-40%
Monocytes	03	2-10%
Eosinophils	02	1-6%
Basophils	00	<1-2%
Platelet count	236	150-410 thou/µL

The biochemical profile showed mild hyponatremia with a serum sodium of 132 mmol/L, while renal function test remained within normal limits, as shown in Table 3.

**Table 3:** Biochemical profile on admission.

Parameter	Values	Range
Serum creatinine	0.98	0.7-1.3 mg/dL
Serum sodium	132	135-145 mmol/L
Serum potassium	3.9	3.50-5.00 mmol/L
Serum chloride	101	95-105 mmol/L

After initial assessment and imaging, the patient was admitted to the intensive care unit (ICU) for closer monitoring, prompted by concerning radiological findings and persistent respiratory symptoms. Empirical antibiotics, maintenance IV fluids, nebulization with bronchodilators, and supportive care were initiated.

A thoracic ultrasound was subsequently performed, which confirmed a moderate left-sided pleural fluid collection with internal echoes suggestive of hemorrhagic content. Coagulation studies and infectious disease screening were requested in preparation for diagnostic pleural tapping and were within acceptable limits, as summarized in Tables 4-5.

**Table 4:** Coagulation profile.

Parameter	Values	Range
Activated partial thromboplastin time (APTT)	25.5	27-40 seconds
APTT control	29.0	24.0-38.0 seconds
Prothrombin time (PT)	14.8	12.70-14.60 seconds
International normalized ratio (INR)	1.10	0.00-1.40 ratio
Mean prothrombin time of control plasma (MNPT)	13.7	<13.7 seconds

**Table 5:** Infectious disease screening results.

Parameter	Values	Range
HIV-1 antibodies	Non-reactive	Non-reactive
HIV-2 antibodies	Non-reactive	Non-reactive
Hepatitis B surface antigen (HbsAg)	Non-reactive	Non-reactive
Hepatitis C Antibodies	Non-reactive	Non-reactive

A diagnostic pleural tap was performed under aseptic conditions, yielding grossly hemorrhagic fluid. While pleural fluid LDH, protein, and hematocrit were not obtained, formal application of Light’s criteria could not be performed. However, the fluid’s grossly hemorrhagic appearance in a clinical context, and absence of bacterial growth or malignant cells supported a hemorrhagic exudate. The results of the pleural fluid analysis are shown in Table 6.

**Table 6:** Pleural fluid analysis.

Test	Result	Interpretation
Appearance	Hemorrhagic	Suggestive of hemothorax
Culture	No growth	No bacterial growth
GeneXpert for TB	Not detected	Negative for tuberculosis DNA
Cytology	Negative	No malignant cells
Acid-fast bacilli smear	Not detected	No acid-fast bacilli seen

As respiratory symptoms worsened, the patient developed mild breathlessness at rest, although oxygen saturation remained stable. An arterial blood gas (ABG) analysis was done, which showed a near-normal pH (7.43), PaCO₂ (37.5 mmHg), bicarbonate (24.7 mmol/L), and lactate (0.7 mmol/L), indicating preserved perfusion. Mild hyponatremia and hypokalemia were also noted, with no requirement for ventilatory or metabolic correction. The ABG findings are denoted in Table 7.

**Table 7:** Arterial blood gas (ABG) analysis findings.

Parameters	Values	Range
pH	7.433	7.350-7-450
pCO_2_	37.5 mmHg	32-48 mmHg
cHCO_3_	24.7 mmol/L	22-26 mmol/L
sO_2_	95%	95-99%
cLac	0.7 mmol/L	0.5-1.6 mmol/L
cNa^+^	128 mmol/L	135-145 mmol/L
cK^+^	3.3 mmol/L	3.5-5.0 mmol/L
cCl^-^	114 mmol/L	98-111 mmol/L
cCa^2+^	1.04 mmol/L	1.15-1.29 mmol/L

**Figure 2:**
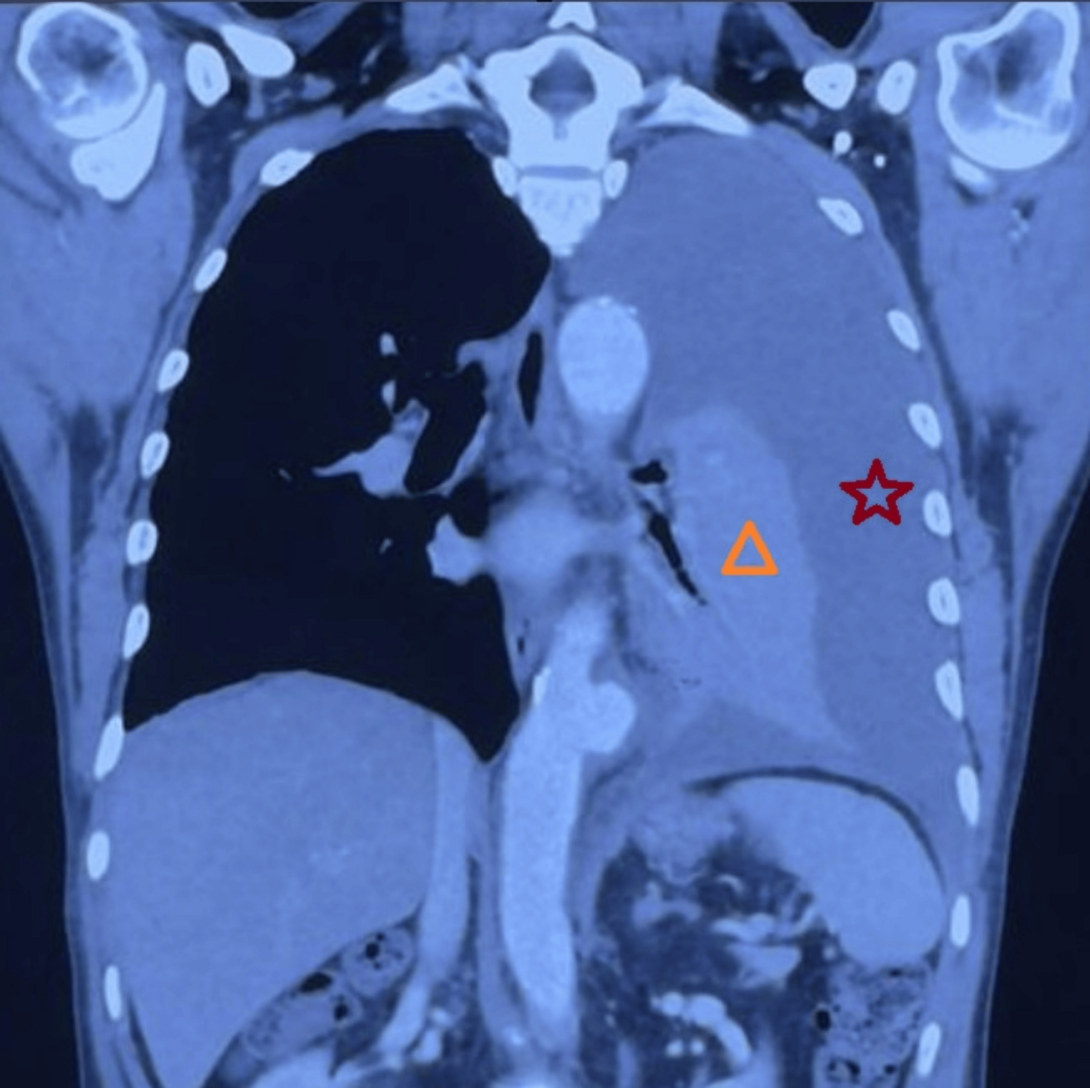
The HRCT denotes a large left-sided pleural effusion (red star) with secondary collapse of the left lung (orange triangle). This coronal CT of the chest shows a large left pleural effusion, which lies between the visceral and the parietal pleura. HRCT, high-resolution computed tomography

**Figure 3:**
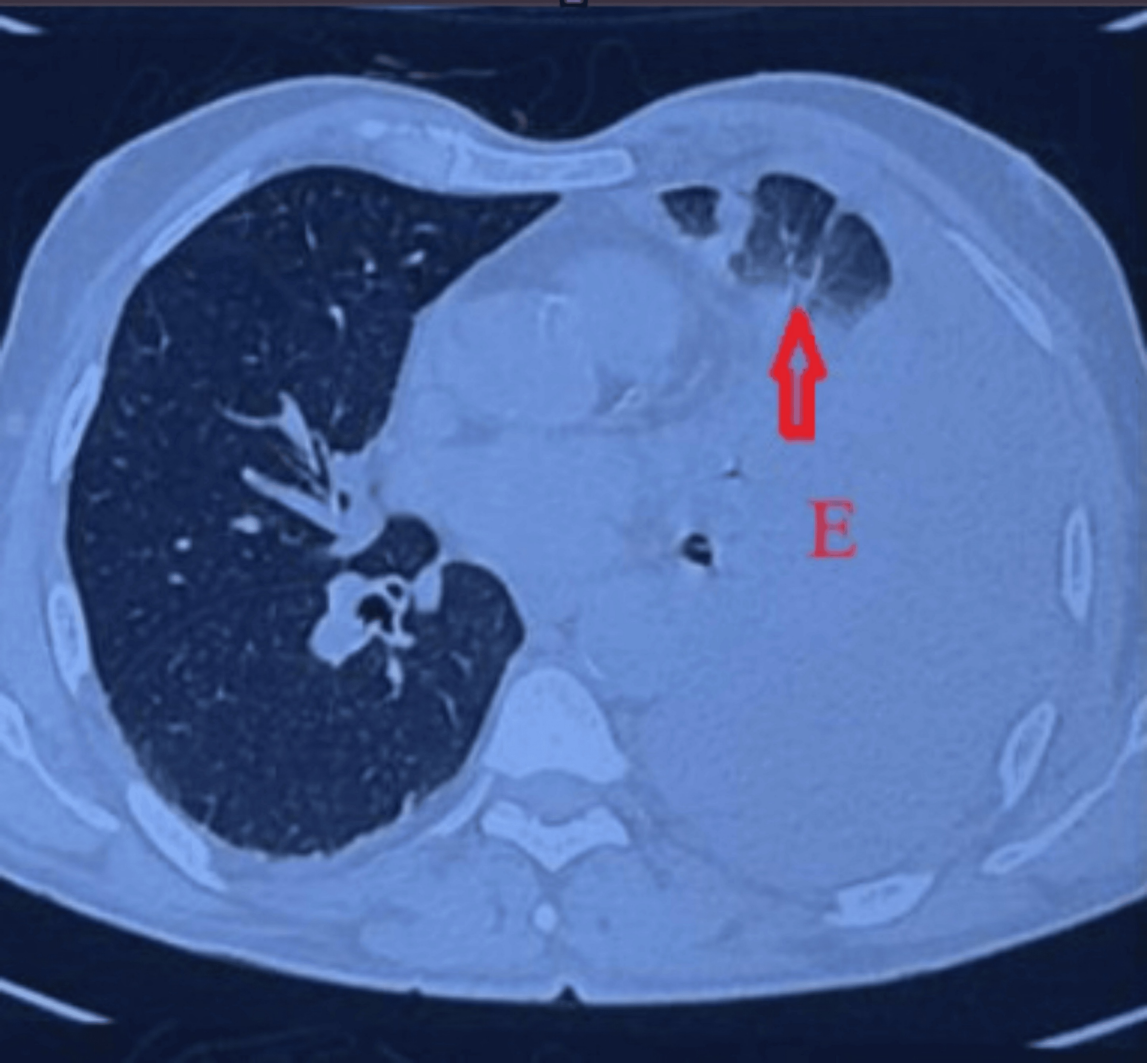
The transverse CT scan on a superior plane shows a left pleural effusion (E) accompanied by collapse of the left lung, as indicated by the arrow.

High-resolution computed tomography (HRCT) of the thorax was performed, which revealed a 54 × 49 mm saccular aneurysm of the descending thoracic aorta (DTA), partially thrombosed and rupturing into the pleural cavity. A large volume of hemothorax with collapse of the left lung was seen. These findings are depicted in Figures 2-3.

**Figure 4:**
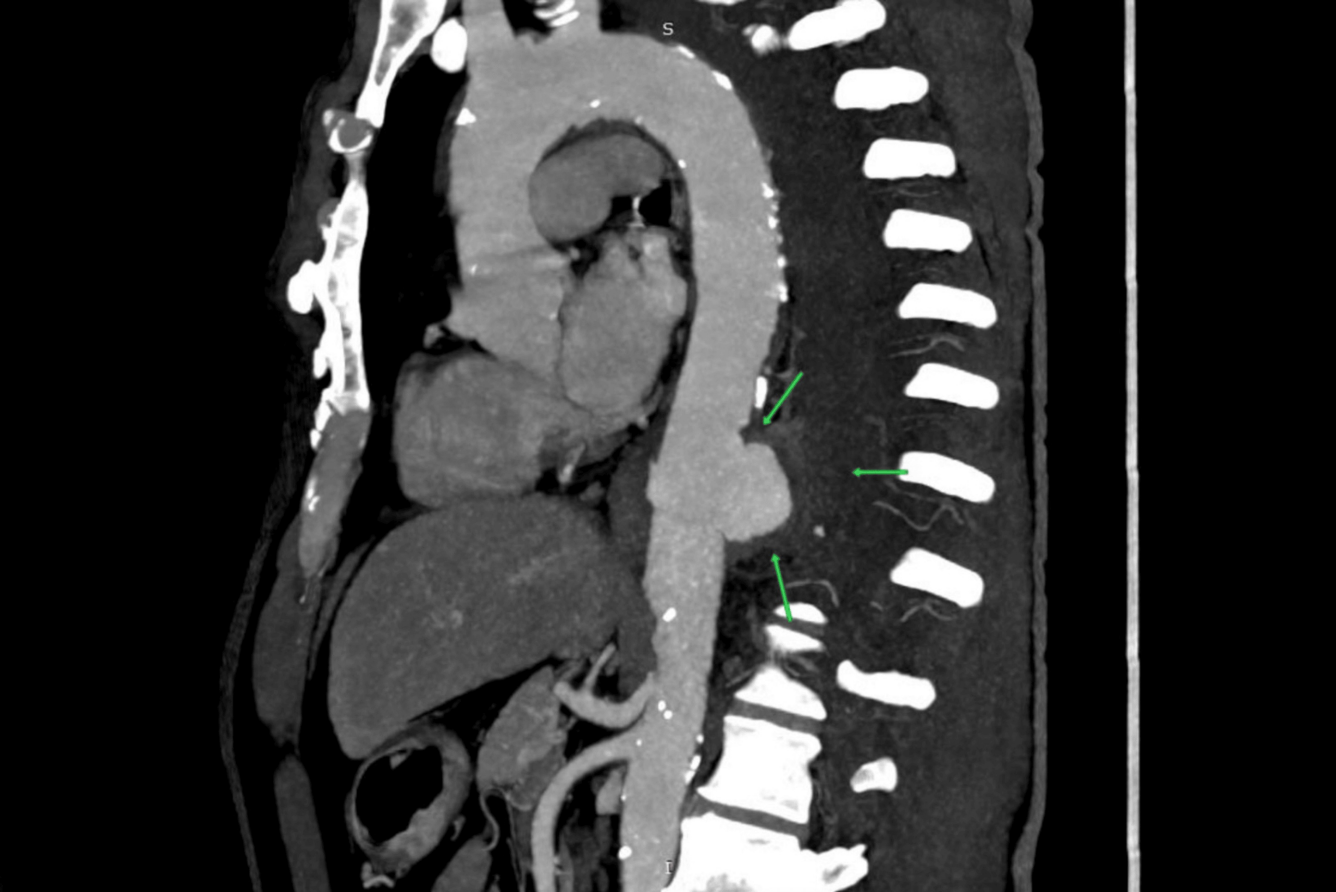
Computed tomography angiography (CTA) showing a saccular aneurysm of the descending thoracic aorta with a contained rupture, as indicated by the arrows.

CTA was subsequently performed for detailed vascular evaluation and procedural planning. It confirmed the contained rupture with no arch or abdominal aorta involvement, and the lesion was deemed suitable for an endovascular repair. CTA imaging is illustrated in Figure 4.

Supportive management was continued in the ICU, including intravenous fluids, empirical antibiotics, nebulized bronchodilators, proton pump inhibitors, and analgesics. Following a multidisciplinary meeting involving cardiovascular surgeons, radiologists, and anesthetists, the patient underwent urgent TEVAR. A 30 × 30 × 120 mm Ankura stent graft (Lifetech Scientific) was selected due to its favorable sealing properties and high conformability. The stent was successfully deployed under general anesthesia via femoral artery access. No anatomical deployment challenges were encountered. Intraoperative fluoroscopic imaging was utilized to ensure accurate placement.

The patient was then shifted back to the ICU for postoperative monitoring. A left-sided intercostal drainage (ICD) tube was inserted, draining approximately 700 mL of altered blood. Supportive therapy included intravenous fluids, proton pump inhibitors, nebulized bronchodilators, empirical antibiotics, and analgesics. He remained hemodynamically stable throughout his ICU stay.

**Figure 5:**
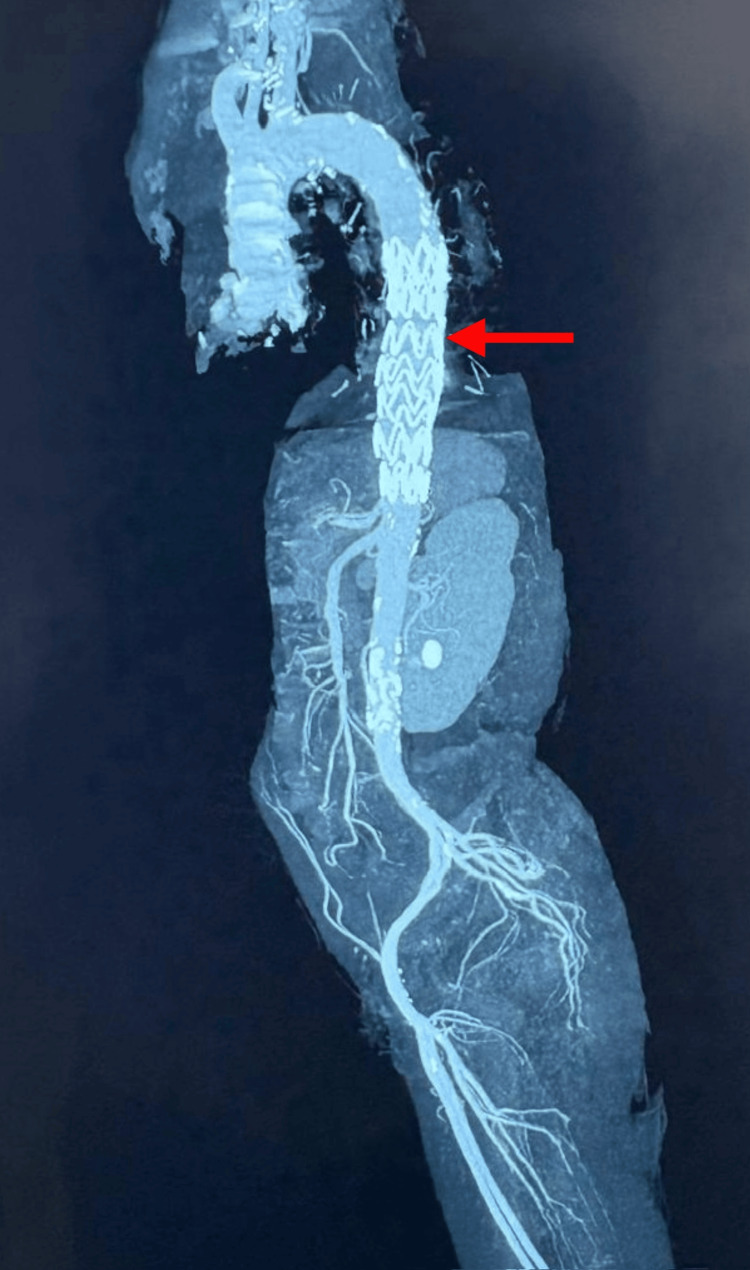
Post-TEVAR CT aortogram (lateral view) illustrating a patent stent graft in the descending thoracic aorta, as indicated by the red arrow. TEVAR, thoracic endovascular aortic repair

A post-procedural CT aortogram was obtained on day 3, which reaffirmed the exclusion of the aneurysm, absence of endoleak, and significant re-expansion of the previously collapsed left lung. These findings are shown in Figure 5.

Post-procedural echocardiography revealed normal biventricular function with a preserved ejection fraction of 55%, concentric left ventricular hypertrophy, and no echocardiographic evidence of pulmonary hypertension.

The patient was discharged on postoperative day 5 in stable condition, with the ICD in situ. Discharge was deemed safe due to complete radiological resolution, stable vitals, and minimal ICD drainage. He was discharged on a regimen consisting of oral antiplatelet agents, a statin, beta-blockers, a calcium channel blocker, a loop diuretic, proton pump inhibitors, and oral antibiotics. He was advised to return for structured follow-up, including ICD review, chest imaging, and surveillance for post-TEVAR complications such as endoleak, graft migration, or reintervention.

**Figure 6:**
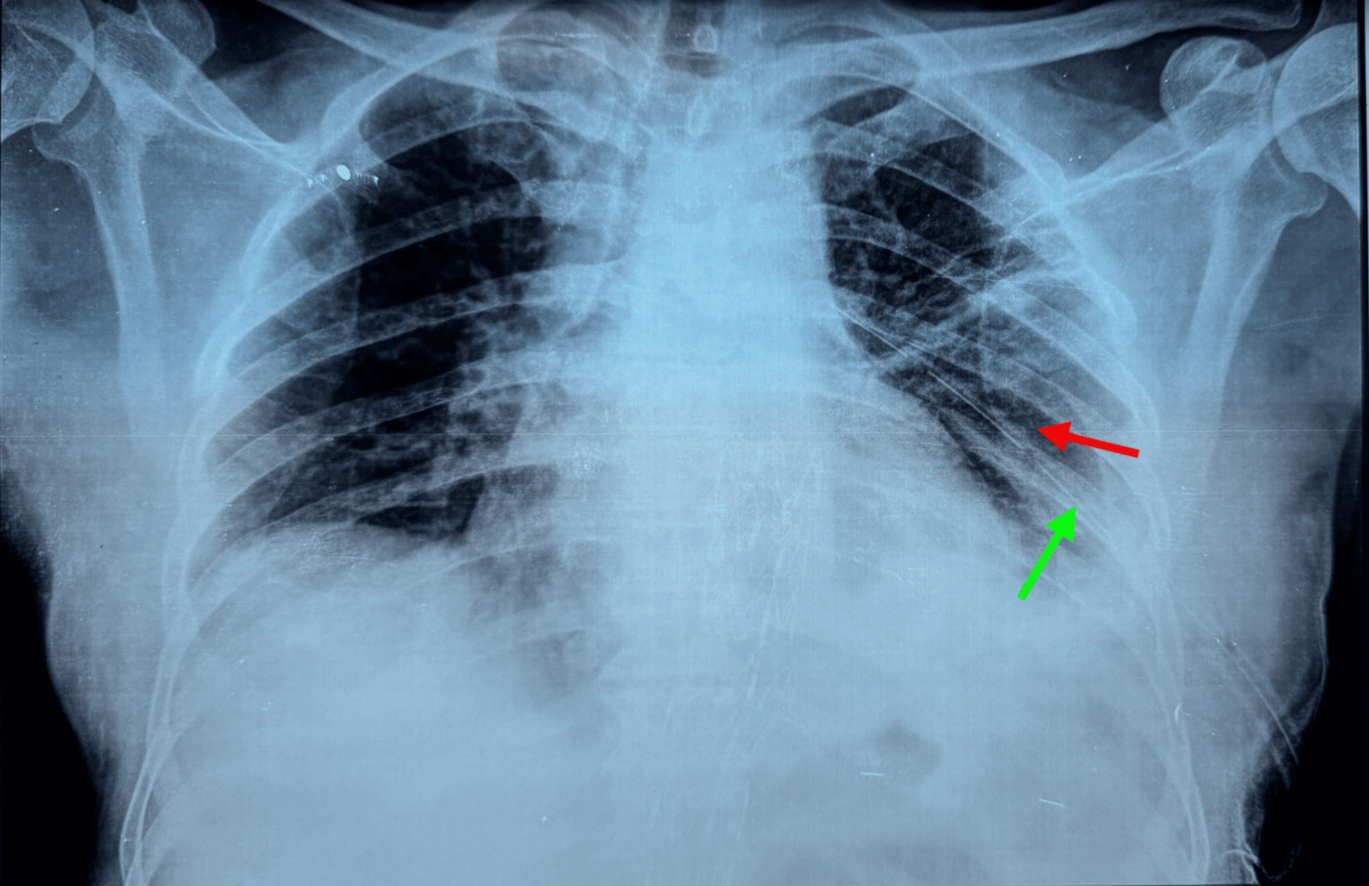
Follow-up chest X-ray showing an intercostal drain in situ (green arrow) and complete resolution of the left hemothorax (red arrow).

At the two-week follow-up, a chest X-ray demonstrated complete resolution of the pleural effusion and full re-expansion of the left lung, as shown in Figure 6. The intercostal drain was removed at this visit without any complications.

**Figure 7:**
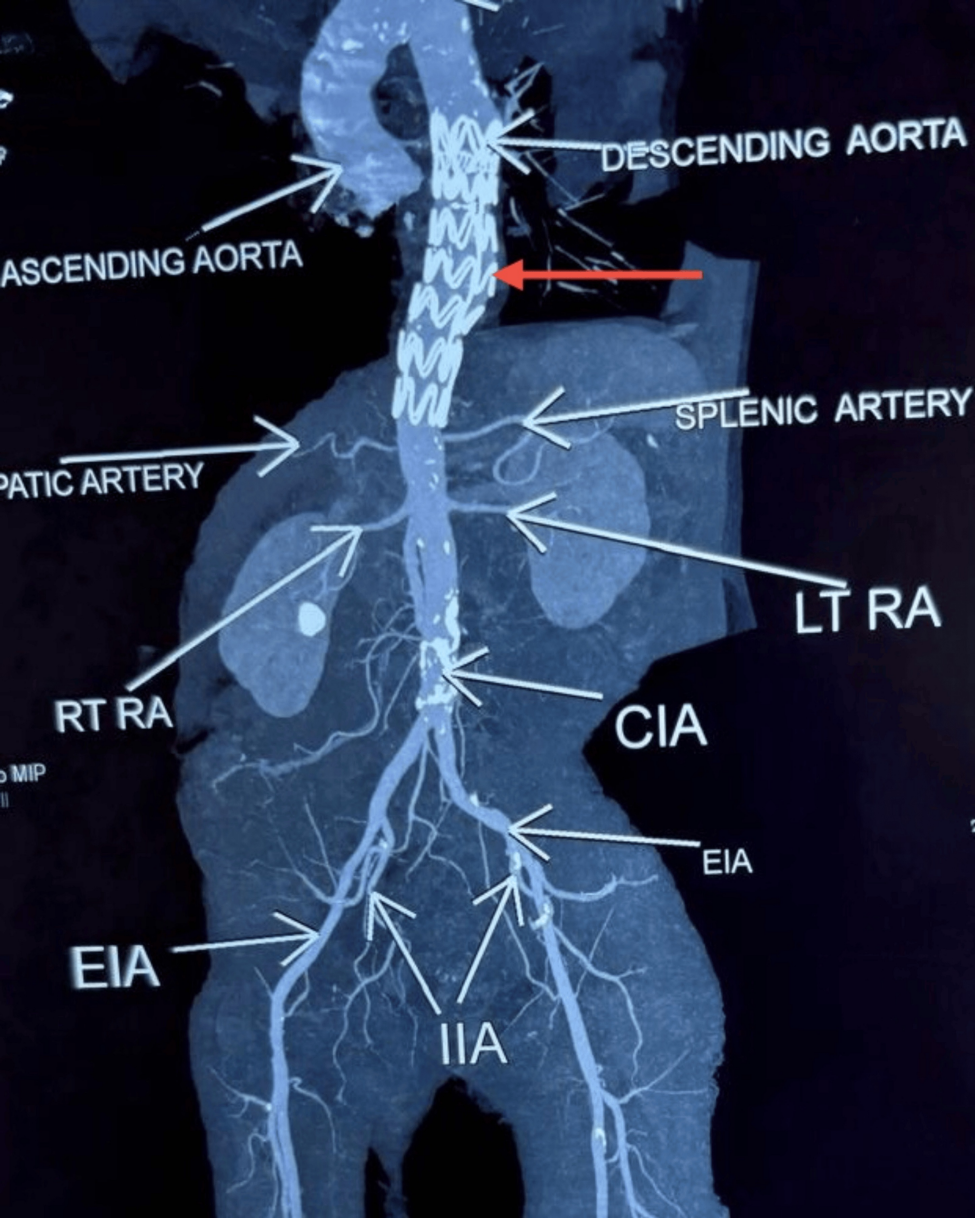
Six-month post-TEVAR CT aortogram showing the Ankura stent graft in the descending thoracic aorta (red arrow) with no evidence of endoleak, graft migration, or recurrent aneurysmal dilatation. The scan confirms stable graft position and preserved perfusion of major visceral and lower limb vessels. TEVAR, thoracic endovascular aortic repair; EIA, external iliac artery; CIA, common iliac artery; RT RA, right renal artery; LT RA, left renal artery

A six-month CT aortogram (Figure 7) confirmed stable graft positioning, absence of endoleak, and no evidence of aneurysmal recurrence.

## Discussion

The contained rupture of a DTAA poses a significant diagnostic challenge due to its varied and frequently misleading clinical presentations, which may resemble cardiopulmonary disorders such as cancer [6]. In our case, the initial misdiagnosis as LRTI resulted in a delay in identifying the underlying aortic pathology, highlighting the importance of a comprehensive diagnostic approach in patients with unexplained respiratory symptoms. The presence of a persistent left-sided pleural effusion, without any signs of infection, necessitated further imaging, which ultimately revealed the underlying vascular cause. Notably, the hemorrhagic fluid yielded by pleural tapping served as an early diagnostic indicator, thereby raising suspicion of a non-infectious etiology.

A significant error in this case was diagnostic anchoring bias, as the patient initially exhibited fever, productive cough, and pleuritic chest pain, which resulted in a premature diagnosis of LRTI. However, the absence of definitive microbiological evidence and the progression of pleural effusion despite antibiotic therapy should have necessitated an earlier reconsideration of the diagnosis. Such misdiagnoses are common in thoracic aortic emergencies, especially when initial symptoms resemble more common cardiopulmonary disorders [7]. This reinforces the need to include aortic pathology in the differential diagnosis when respiratory symptoms evolve, and pleural fluid accumulates without a definitive cause. In our case, differential diagnoses such as tuberculosis and malignancy were considered but were deemed unlikely due to the acute symptom onset, absence of constitutional signs, lack of lymphadenopathy, and negative pleural fluid analysis for acid-fast bacilli and Gene Xpert.

The hemodynamic stability observed in this instance of a contained DTAA rupture is unusual and contradicts standard assumptions, as the majority of aorta ruptures are linked to swift circulatory failure. In rare cases, regional tamponade may temporarily stabilize the patient, concealing the life-threatening vascular emergency. This illusory stability may delay diagnosis, particularly when clinical and radiological characteristics resemble cardiovascular or neoplastic disorders. This underscores the need for heightened vigilance, since delayed identification may result in abrupt and lethal deterioration [8].

Imaging is highly essential for differentiating DTAA rupture from other thoracic emergencies. The chest X-ray indicated a left-sided pleural effusion, while the notable rightward tracheal deviation served as an early radiological indicator of mass effect, potentially suggesting a vascular rupture. In this case, the aneurysm's position in the DTA, situated posterior to the cardiac silhouette, resulted in its non-visualization on the chest X-ray.

HRCT is frequently utilized in the first diagnostic phase when respiratory symptoms progress despite intervention, as it offers superior detail of lung parenchyma and pleural disease [9]. In this case, HRCT initially suggested vascular pathology by identifying a saccular aneurysm accompanied by a massive hemothorax with lung collapse. This warranted additional assessment with CT angiogram (CTA), recognized as the gold standard for evaluating thoracic aortic illness due to its capacity to distinctly visualize the aortic lumen and signs of active extravasation [10]. In our patient, CTA confirmed the localized rupture and supplied critical anatomical information for operative preparation. Despite the availability of modern imaging techniques, thoracic aortic emergencies are often misdiagnosed, especially when symptoms mimic more prevalent cardiopulmonary conditions, a tendency observed in extensive multicenter studies [11].

This case highlights the need for timely and appropriate imaging. Although chest X-ray was the first investigation performed, it was the HRCT and CTA that provided definitive diagnostic clarity. The combination of cross-sectional imaging allowed for quick identification of the aneurysm, appropriate surgical planning, and a timely intervention. Clinically, this case suggests that early use of CT angiography should be considered in patients with unexplained or hemorrhagic pleural effusion when clinical, laboratory, and imaging findings are discordant.

The management of DTAA includes both open surgical and TEVAR. Although TEVAR has emerged as the favored choice in numerous instances owing to its minimally invasive characteristics, open repair continues to be a significant alternative, especially for younger patients, individuals with connective tissue problems, or when anatomical conditions are incompatible with endovascular methods. Open surgery provides enhanced long-term durability and may correlate with reduced reintervention rates in specific instances [12]. 

TEVAR offers a less invasive option for aortic repair, markedly decreasing perioperative morbidity and facilitating fast aortic stability, especially in high-risk patients [13]. TEVAR was performed effectively in our patient, resulting in total aneurysm exclusion without any complications. Continuous post-procedural monitoring is essential after TEVAR, since patients are susceptible to delayed consequences, including endoleaks, graft migration, and aneurysmal progression, all of which require prompt reintervention [14]. Ensuring compliance with long-term follow-up protocols is crucial, as inadequate adherence has been linked to missed diagnosis of problems, possibly jeopardizing patient outcomes [15].

Structured post-TEVAR surveillance includes CT imaging at 1, 6, and 12 months, followed by annual imaging [16]. Blood pressure is strictly controlled to remain below 120/80 mmHg, typically with beta-blockers or ACE inhibitors, to reduce shear stress and prevent aneurysmal progression. In our patient, a six-month follow-up CT aortogram confirmed successful aneurysm exclusion.

While formal screening programs for thoracic aortic aneurysms are not extensively implemented, opportunistic screening in high-risk individuals, such as those with chronic hypertension, smoking history, or incidental mediastinal widening observed on chest X-rays, may enable earlier detection and intervention [17].

A multidisciplinary team comprising emergency physicians, radiologists, intensivists, and cardiovascular surgeons was central to this patient's outcome. Their collaborative approach enabled early reconsideration of diagnosis, expedited vascular imaging, and seamless transition to definitive treatment.

Our case adds to the limited literature by demonstrating the unusual presentation of a hemodynamically stable, contained rupture of a DTAA, which closely resembled a LRTI. While prior reports highlight acute instability in ruptured DTAA, our patient’s stability contributed to the diagnostic delay. This case reinforces the importance of considering aortic pathology in patients with unexplained pleural effusion and deteriorating respiratory symptoms, especially when conventional diagnostic investigations yield equivocal results. In contrast to the dramatic presentation of acute rupture, confined DTAA ruptures may resemble infectious disorders, resulting in misdiagnosis and delays in treatment. Clinicians must identify subtle warning indications before the aneurysm advances to a catastrophic rupture, facilitating early imaging and prompt management.

## Conclusions

This case highlights the diagnostic challenges in DTAA rupture, emphasizing that hemodynamic stability does not exclude life-threatening pathology. Atypical symptoms resembling LRTI may mislead clinicians, causing diagnostic delays and missed opportunities for timely intervention. Early use of advanced imaging, such as HRCT and CTA, is crucial, especially when clinical and radiological findings diverge. In this case, initial misdiagnosis led to a stepwise escalation of investigations: chest X-ray revealed pleural effusion, ultrasound showed hemorrhagic content, HRCT identified a saccular aneurysm, and CTA confirmed a contained rupture. Each modality contributed to diagnostic refinement and guided definitive management. While TEVAR was successful, outcomes vary with anatomical complexity and comorbidities, underscoring the need for individualized planning. Long-term surveillance includes CT angiography at 1, 6, and 12 months, then annually, along with strict blood pressure control. This case reinforces the need for a high index of suspicion for aortic pathology in patients with unexplained pleural effusion and non-resolving respiratory symptoms and supports earlier use of CTA in atypical presentations.
